# Breakpoints for the Classification of Anti-*Candida* Compounds in Antifungal Screening

**DOI:** 10.1155/2021/6653311

**Published:** 2021-04-06

**Authors:** Danielle da Nóbrega Alves, Alana Rodrigues Ferreira, Allana Brunna Sucupira Duarte, Ana Karoline Vieira Melo, Damião Pergentino de Sousa, Ricardo Dias de Castro

**Affiliations:** ^1^Department of Clinic and Social Dentistry, Graduate Program in Natural and Synthetic Bioactive Products (PgPNSB), Center for Health Sciences, Federal University of Paraiba, João Pessoa, PB, Brazil; ^2^Graduate Program in Natural and Synthetic Bioactive Products (PgPNSB), Center for Health Sciences, Federal University of Paraíba, João Pessoa, PB, Brazil; ^3^Graduate Program in Dentistry (PPGO), Center for Health Sciences, Federal University of Paraiba, João Pessoa, PB, Brazil; ^4^Department of Pharmaceutical Sciences, Center for Health Sciences, Federal University of Paraíba, João Pessoa, PB, Brazil

## Abstract

**Introduction:**

The absence of a standardized classification scheme for the antifungal potency of compounds screened against *Candida* species may hinder the study of new drugs. This systematic review proposes a scheme of interpretative breakpoints for the minimum inhibitory concentration (MIC) of bioactive compounds against *Candida* species in *in vitro* tests.

**Materials and Methods:**

A literature search was conducted in the PubMed, Scopus, Web of Science, Lilacs, and SciFinder databases for the period from January 2015 to April 2020. The following inclusion criterion was used: organic compounds tested by the microdilution technique according to the Clinical and Laboratory Standards Institute protocol against reference strains of the genus *Candida*. A total of 545 articles were retrieved after removing duplicates. Of these, 106 articles were selected after applying the exclusion criteria and were evaluated according to the number of synthesized molecules and their chemical classes, the type of strain (reference or clinical) used in the antifungal test, the *Candida* species, and the MIC (in *μ*g/mL) used.

**Results:**

The analysis was performed based on the median, quartiles (25% and 75%), maximum, and minimum values of four groups: all strains, ATCC strains, *C. albicans* strains, and *C. albicans* ATCC strains. The following breakpoints were proposed to define the categories: MIC < 3.515 *μ*g/mL (very strong bioactivity); 3.516-25 *μ*g/mL (strong bioactivity); 26-100 *μ*g/mL (moderate bioactivity); 101-500 *μ*g/mL (weak bioactivity); 500-2000 *μ*g/mL (very weak bioactivity); and >2000 *μ*g/mL (no bioactivity).

**Conclusions:**

A classification scheme of the antifungal potency of compounds against *Candida* species is proposed that can be used to identify the antifungal potential of new drug candidates.

## 1. Introduction

The increased prevalence of serious infections caused by *Candida* species has driven the search for new molecules with antifungal effects and potential for clinical use [1]. Because they are commensal, these microorganisms are especially associated with infections in immunocompromised individuals, resulting in problems that are difficult to solve and require adequate therapy for successful treatment [2].

Few antifungal drugs are available, and the continuous emergence of resistant strains is the greatest challenge related to treatment [3]. The main drugs used to treat fungal infections belong to the classes of polyenes (amphotericin B), pyrimidines (5-fluorocytosine), azoles (fluconazole, voriconazole, itraconazole, etc.), and echinocandins (caspofungin and micafungin) [4]. The main resistance mechanisms include drug efflux pumps, decreased cell permeability to drugs, alteration of the drug-binding site, and biofilm formation, which hinder drug permeability. Resistance to azoles and echinocandins is frequently reported in the scientific literature [5].

Clinical trials elucidating the effects of new antifungal drugs are still scarce. Some clinical studies have assessed the associations of drugs that are already recognized and available on the market [6, 7]. Combinations of these drugs represent strategies for achieving pharmacological synergism and promoting the treatment of persistent infections. To evaluate new treatments for infections caused by *Candida* species, investigations generally consider the use of drugs already known to be effective in groups of people with specific morbidity profiles, such as premature infants [8] and individuals with clinical features of recurrent and severe infections [9, 10].

The synthesis of antifungal compounds is an important strategy for obtaining new antifungal drugs with efficacy similar or superior to that observed for available drugs. The discovery of new therapeutic agents with different mechanisms of action allows us to broaden the spectrum of action against resistant microorganisms. In general, scientific studies aimed at synthesizing new antifungal agents evaluate the effects of these molecules using different methodological protocols, which include initial studies to determine their minimum inhibitory concentrations (MICs) in agar diffusion and broth microdilution tests. Often, these protocols are modified in terms of cell concentrations, the type of culture medium, and the microorganisms used, among others. These variations complicate interpretation of the results and advances in knowledge about the effects of compounds on fungal cells.

This difficulty indicates that research on new drugs followed by antifungal evaluations would benefit from the establishment of standardized methods to enable comparisons and further knowledge gains. The establishment of breakpoints to classify the antifungal potential of new compounds is essential to guide the performance of new synthesis protocols and/or antifungal evaluation assays. However, the scientific literature does not include a proposal for the definition of breakpoints for new organic molecules. Often, researchers classify the antifungal potential of new compounds based on an empirical analysis of results obtained for MICs. Available guidelines proposed by the Clinical and Laboratory Standards Institute (CLSI) [11] and the European Committee on Antimicrobial Susceptibility Testing (EUCAST) [12] offer parameters for the classification of substances intended for clinical use. However, breakpoints that can be applied in the search for new molecules with potential for *in vivo* clinical evaluations and in substance improvement to achieve its best biological profile must be established, especially in antimicrobial screening. The objective of this study is to propose a classification scheme of the antifungal potency of new compounds with activity against *Candida*.

## 2. Methods

### 2.1. Study Question

This systematic review was carried out to address the specific question, “What are the MIC breakpoints for interpretation of the anti-*Candida* activity of new organic molecules?”

### 2.2. Eligibility Criteria

Systematic screening of articles was performed by four independent examiners according to the following inclusion criteria.

#### 2.2.1. Biological Activity

The article examined the effects of synthetic molecules with anti-*Candida* (reference ATCC) activity based on their MIC values (*μ*g/mL) obtained by the microdilution technique. To maintain standardization in the analysis, grouped MIC values (e.g., MIC50, MIC60, or MIC90) were not included.

#### 2.2.2. Study Design

The article reported an *in vitro* study evaluating antifungal activity against *Candida* species.

#### 2.2.3. Methodological Quality

The article utilized methods recommended by the CLSI to determine MICs using the microdilution technique (studies using a growth inhibition zone/disc diffusion assay were not included). Study designs, sample representativeness, validity, reproducibility, losses, biases, the accuracy of methods, and outcomes were carefully investigated [13].

#### 2.2.4. Language

No restriction on the language of publication was applied.

### 2.3. Exclusion Criteria

1. The study tested organic molecules available for clinical use with improved physical and chemical properties, such as those in the form of nanoparticles and nanocomposites
2. The study did not precisely define MIC values
3. The results are reported in molarity values

### 2.4. Search Strategy and Study Selection

This study followed the Preferred Reporting Items for Systematic Reviews and Meta-Analyses (PRISMA) guidelines for systematic reviews [14, 15]. Four examiners conducted the search independently in the PubMed, Scopus, Web of Science, Lilacs, and SciFinder databases. Articles published from January 2015 to April 2020 were searched comprehensively, with no restrictions on the publication language. The MeSH terms used for the search were “agents, antifungal,” “therapeutic fungicides,” “fungicidi,” “fungicidie,” “fungico,” “fungicos,” “antifungal agents,” “antifungal,” “therapeutic,” “antibiotics, antifungal,” “antibiotics, antifungals,” “antibiotics, antimycotics,” “antifungal antibiotics,” “therapeutic,” “fungicides chemistry,” “fungicides/therapeutic,” “fungicides, industrial,” “fungicides, industrial,” “industrial fungicides,” “*Candida*,” “*Candida*/*albicans*,” “*Candida*/analysis,” “*Candida*/chemistry,” “*Candida*/drug effects,” “*Candida*/infection,” “*Candida* albicans,” “*Candida albicans*/analysis,” “*Candida albicans*/chemistry,” “chemical synthesis,” “synthesis techniques,” “Synthesis Technique, Organic,” “Synthesis Techniques, Organic,” “Technique, Organic Synthesis,” “Techniques, Organic Synthesis,” “Methods of Organic Synthesis,” “Organic Synthesis Methods,” “Method, Organic Synthesis,” “Methods, Organic Synthesis,” “Organic Synthesis,” “Organic Syntheses,” “Syntheses, Organic,” “Synthesis, Organic.” Boolean operators “AND” and “OR” were used to combine the search terms (Table 1).

After searching the databases, the articles retrieved were imported into EndNote X9, and duplicate articles were removed. Full texts were read after applying the eligibility criteria in cases where the titles and abstracts did not allow exclusion. A third examiner resolved any disagreement between the two reviewers. The data were extracted into an individual spreadsheet to gather the following information: the quantities and chemical classes of the molecules, the type of strain (reference or clinical), the type of *Candida* species, and the MIC.

### 2.5. Data Analysis

The data from the studies were analyzed based on MIC values (*μ*g/mL) and classified according to descriptive statistical parameters; quartiles were calculated using Excel 365.

## 3. Results

**Figure 1:**
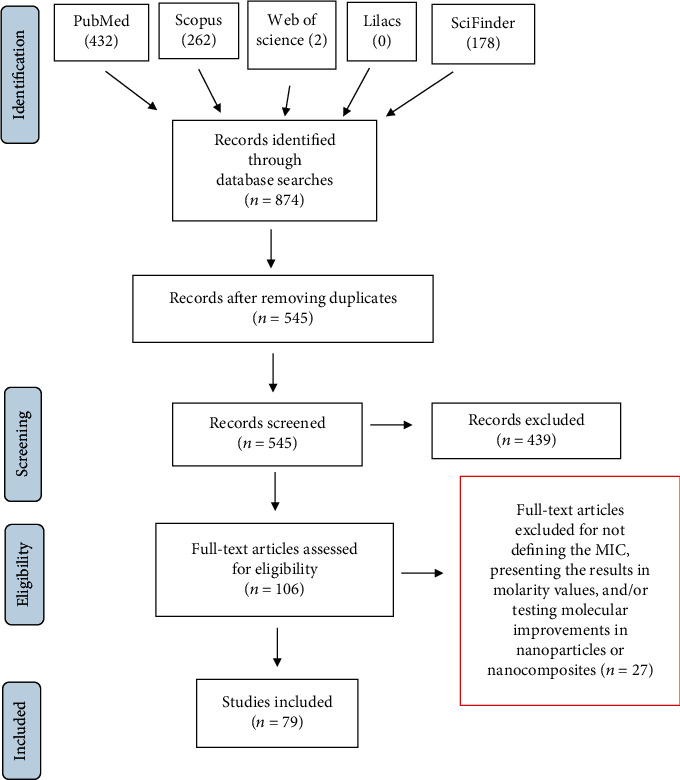
Flowchart of the bibliographic search according to PRISMA guidelines. The first exclusion step targeted studies that did not meet the eligibility criteria, i.e., studies unrelated to the synthesis of organic molecules that were tested according to the CLSI protocol against the reference strains of the genus *Candida*. After reading the full texts, we excluded studies that did not precisely report the MICs (three studies), studies presenting the results in molarity values (18 studies), and/or studies testing particle improvements in either nanoparticles (five studies) or nanocomposites (one study).

A total of 874 articles were identified, and after removing duplicates, 545 documents remained. After reading the full articles, 106 studies were selected, while those that were unrelated to the synthesis of organic compounds and their testing according to the CLSI protocol against the reference strains of the genus *Candida* were excluded. Studies were also excluded for not precisely reporting MICs (*n* = 3 studies), presenting the results in molarity values (*n* = 18 studies), or using formulations in the form of either nanoparticles (*n* = 3 studies) or nanocomposites (*n* = 1 study). We additionally excluded studies combining these characteristics, such as those testing compounds in the form of nanoparticles and presenting the results as molarity values (*n* = 1 study) and those testing molecules in the form of nanoparticles without defining the precise value of the MIC (*n* = 1 study). Thus, a total of 27 studies were excluded from the analysis among the 106 articles selected in the previous step. From the remaining studies, 1046 evaluated molecules were considered. The selection process according to PRISMA recommendations is depicted in the flowchart shown in Figure 1 [15].

Table 2 shows the MIC values (*μ*g/mL) of the substances eligible for this study. Because the data did not present a normal distribution, they were organized according to descriptive statistical measures: the median, quartiles (25% and 75%), and the maximum and minimum values. This analysis was performed in four groups: group 1, all strains (*n* = 2199); group 2, all ATCC strains (*n* = 2005); group 3, all *C. albicans* strains (*n* = 1147); and group 4, all *C. albicans* ATCC strains (*n* = 969).

Different descriptive analyses yielded similar values. Based on the data above, a classification of the MICs of synthetic compounds into six categories was proposed: very strong bioactivity, strong bioactivity, moderate bioactivity, weak bioactivity, very weak bioactivity, and no bioactivity (Table 3).

The first- and second-quartile values of the MICs of substances with activity against *C. albicans* ATCC strains and all ATCC strains were higher than those of substances with activity against all strains. Thus, to cover substances with the best results against strains requiring higher MIC values, the categories in Table 3 were defined based on the highest MIC values found. In addition, after observing that the third quartile covered a very wide interval (MICs greater than 100 *μ*g/mL and less than 2000 *μ*g/mL), we further split this quartile into molecules with MICs between 100 and 500 *μ*g/mL, which were classified as weak, and molecules with MICs between 500 and 2000 *μ*g/mL, which were classified as very weak. This split prevented the inclusion of substances with an up to 20-fold MIC difference in the same category.

The consistency of the proposed classification can be demonstrated based on an MIC analysis of molecules such as fluconazole, itraconazole, voriconazole, amphotericin B, and caspofungin because they have been used as positive controls in several studies [16–20] and would be classified as very strong according to Table 3. The above classification was proposed based only on the MIC values of organic molecules; therefore, this classification should not be the only parameter considered in the analysis of a substance or drug as other relevant aspects should be considered, such as toxicity, cost, and availability.

All articles analyzed were classified as *in vitro* studies published from January 2015 to April 2020 and met the inclusion criteria. Table 1S (supplementary material) describes the characteristics of and the data collected from the selected studies.

## 4. Discussion

Compared to the development of other drugs, the design of antifungal drugs is more complex because fungi are eukaryotes, and many of the potential targets for therapy are also found in humans, resulting in a higher risk of toxicity [21]. Thus, the search for new chemical agents with high antimicrobial activity is incessant because the development of new antimicrobial drugs will expand the therapeutic arsenal available to the population, especially for the treatment of infections resistant to currently available drugs [22].

The protocols available to evaluate the antifungal activity of chemicals include the guidelines proposed by the CLSI [11] and EUCAST [12], both of which adopt the broth microdilution method but have some methodological differences and are recognized as independent proposals for the interpretation of fungal sensitivity. The CLSI guidelines are widely applied in the USA and used by the Food and Drug Administration for approval of new drugs in the country. The EUCAST guidelines were developed to standardize MIC breakpoints throughout Europe [23, 24]. However, several other methods can also be used to determine antifungal susceptibility, such as disc diffusion, stainless steel cylinders, well agar diffusion, and macrodilution [25]. This diversity of methods adopted by researchers had led to considerable variation in how results are reported, which complicates standardization and the selection of products in which more resources should be invested.

From the analysis of the selected studies, we found that most of the evaluated strains used in the studies are reference strains, which is very important for the evaluation of the research results because reference strains are used in product testing, as positive and negative controls, as indicator organisms, or as identification standards, thus ensuring the quality of laboratory activities in microbiological analyses [26, 27].

The CLSI and EUCAST provide breakpoints for antifungal agents already used in clinical practice, i.e., they provide acceptable MIC values for the treatment of fungal infections caused by *Candida species* [11, 12, 28]. Regarding organic compounds with antifungal potential, the literature does not present a standardized classification for the interpretation of their bioactivity results, which may hinder accurate evaluations of the antifungal potential of new compounds as well as the identification of potentially promising structures. In addition, MIC values considered high for a new molecule may point to new chemical modifications to the substance that may increase its pharmacological potency. In addition to the verification of MIC values, the determination of the minimum fungicidal concentration (CFM) contributes to a better understanding of the antifungal activity profiles of compounds. The discovery of new drugs with fungicidal activity can improve the treatment of immunocompromised individuals with persistent and serious infections [4].

The proposal of interpretive breakpoints is important for the determination of the antifungal potency of new organic compounds. Thus, in studies aimed at identifying candidates for antifungal drugs, as well as in screens against *Candida* species, this classification will be decisive for the selection of chemical structures with the best bioactivity, the structural groups responsible for antifungal activity (pharmacophoric groups), or structural groups that potentiate bioactivity or promote inactivity; this information will support the search for substances with high pharmacological potency [29].

Some studies have proposed breakpoints for the activity of plant extracts against *Candida* species [30–33]. However, considering the complexity of the chemical compositions of these products, the results obtained in evaluations of pure compounds should be interpreted based on other criteria, and new studies must be performed to establish a classification of breakpoints for complex mixtures of natural products. Popiolek et al. [34] proposed a classification of the antifungal bioactivity of 3-hydroxy-2-naphthohydrazide derivatives against a number of microorganisms, such as bacteria, filamentous fungi, and yeasts, including *Candida* species. In their proposed classification, no bioactivity was defined by an MIC > 1000 *μ*g/mL; mild bioactivity was defined by an MIC of 501-1000 *μ*g/mL, moderate bioactivity was defined by an MIC of 126-500 *μ*g/mL, good bioactivity was defined by an MIC of 26-125 *μ*g/mL, strong bioactivity was defined by an MIC of 10-25 *μ*g/mL, and very strong bioactivity was defined by an MIC < 10 *μ*g/mL. Although this classification was proposed for synthetic molecules, the authors did not indicate the criteria used to establish the categories. Therefore, we consider establishing breakpoints based on global research investigating the activity of new compounds against *Candida* species with standardization of the methods and fungal strains adopted to be important.

As recommended by CLSI protocols [5], the proposal of breakpoints in this study was based on the MIC values (*μ*g/mL) of organic molecules with anti-*Candida* activity determined using the microdilution technique. Based on an analysis of 106 published scientific articles involving 1046 molecules, six classification categories are proposed (very strong bioactivity, strong bioactivity, moderate bioactivity, weak bioactivity, very weak bioactivity, and no bioactivity), which expand the analysis of the results and suggest compounds with potential for use in new drugs, especially those with MIC values ≤ 100 *μ*g/mL. Notably, however, the molecular structures of compounds with high MIC values can be chemically modified to improve pharmacological potency.

We analyzed groups of molecules based on the type of fungal strain studied (all strains, ATCC strains, *C. albicans* strains, and *C. albicans* ATCC strains). The studies reported results mainly for the following species: *C. krusei*, *C. parapsilosis*, *C. glabrata*, *C. tropicalis*, and *C. utilis.* These species are often selected because they are associated with infectious processes in humans and have virulence factors associated with greater disease severity [35–37]. Stratification of the results by the type of strain contributes to a better understanding of such results and to the establishment of breakpoints considering the diversity of findings for different situations.

## 5. Conclusions

MIC analysis of hundreds of antifungal organic compounds allowed the establishment of a classification scheme of their inhibitory potency against *Candida* species, which will serve as a reference to identify new antifungal compounds and determine their levels of antimicrobial activity. The present proposal will overcome the lack of an efficient classification system to assist in the discovery of candidate compounds for anti-*Candida* drugs.

## Figures and Tables

**Table 1:** Search strategies in the bibliographic databases used to retrieve the articles for this systematic review.

Database (since 2015)	Search strategy (descriptors and combinations with Boolean operators)
PubMed	#1 search: (((((((((((((((((((“agents, antifungal”) OR (“therapeutic fungicides”)) OR (“fungicidi”)) OR (“fungicidie”)) OR (“fungico”)) OR (“fungicos”)) OR (“antifungal agents”)) OR (“antifungal”)) OR (“therapeutic”)) OR (“antibiotics, antifungal”)) OR (“antibiotics, antifungals”)) OR (“antibiotics, antimycotics”)) OR (“antifungal antibiotics”)) OR (“therapeutic”)) OR (“fungicides chemistry”)) OR (“fungicides/therapeutic”)) OR (“fungicides, industrial”)) OR (“fungicides, industrial”))) OR (“industrial fungicides”). Filters: full texts in the last 5 years #2 search: ((((((((“candida”) OR (“candida/albicans”)) OR (“candida/analysis”)) OR (“candida/chemistry”)) OR (“candida/drug effects”)) OR (“candida/infection”)) OR (“candida albicans”)) OR (“candida albicans/analysis”)) OR (“candida albicans/chemistry”). Filters: full texts in the last 5 years #3 search: ((((((((((((((“chemical synthesis”) OR (“synthesis techniques”[All Fields])) OR (“Synthesis Technique, Organic”)) OR (“Synthesis Techniques, Organic”)) OR (“Technique, Organic Synthesis”)) OR (Techniques, Organic Synthesis)) OR (“Techniques, Organic Synthesis”)) OR (“Methods of Organic Synthesis”)) OR (“Organic Synthesis Methods”)) OR (“Method, Organic Synthesis”)) OR (“Methods, Organic Synthesis”)) OR (“Organic Synthesis”)) OR (“Organic Syntheses”)) OR (“Syntheses, Organic”)) OR (“Synthesis, Organic”). Filters: full texts in the last 5 years #1 AND #2 AND #3

Scopus	#1 search: (ALL (“chemical synthesis” OR “Method, Organic Synthesis” OR “Synthesis Technique, Organic” OR “Synthesis Techniques, Organic” OR “Technique, Organic Synthesis” OR “Techniques, Organic Synthesis”) OR ALL (“Methods of Organic Synthesis” OR “Organic Synthesis Methods” OR “Methods, Organic Synthesis” OR “Organic Synthesis” OR “Organic Syntheses” OR “Syntheses, Organic” OR “Synthesis, Organic”)) AND DOCTYPE (ar) AND PUBYEAR > 2015 #2 search: (ALL (“candida”) OR ALL (“candida/albicans”) OR ALL (“candida tropicalis”) OR ALL (“candida glabrata”) OR ALL (“candida albicans”) OR ALL (“candida/analysis”) OR ALL (“candida chemistry”) OR ALL (“candida/drug effects”) OR ALL (“candida infection”) OR ALL (“candida albicans/analysis”)) AND DOCTYPE (ar) AND PUBYEAR > 2015 #3 search: (ALL ((“Agents,antifungal” OR “antifungal agents” OR “antifungal antibiotics” OR “antibiotics,antifungal” OR “fungicides,therapeutic” OR “therapeutic fungicides” OR “fungicides industrial”)) AND DOCTYPE (ar) AND PUBYEAR > 2015) #1 AND #2 AND #3

Web of Science	#1 search: (TS = (“agents, antifungal” OR “antifungal agents” OR “antifungal antibiotics” OR “antibiotics, antifungal” OR “fungicides, therapeutic” OR “therapeutic fungicides” OR “fungicides industrial”)) *AND* DOCUMENT TYPES: (Article) Indexes = SCI-EXPANDED, SSCI, A&HCI, CPCI-S, CPCI-SSH, ESCI Tempo estipulado = 2015-2020 #2 search: (TS = (“chemical synthesis” OR “Synthesis Technique, Organic” OR “Synthesis Techniques, Organic” OR “Technique, Organic Synthesis” OR “Techniques, Organic Synthesis” OR “Methods of Organic Synthesis” OR “Organic Synthesis Methods” OR “Method, Organic Synthesis” OR “Methods, Organic Synthesis” OR “Organic Synthesis” OR “Organic Syntheses” OR “Syntheses, Organic” OR “Synthesis, Organic”)) *AND* DOCUMENT TYPES: (Article) Indexes = SCI-EXPANDED, SSCI, A&HCI, CPCI-S, CPCI-SSH, ESCI Tempo estipulado = 2015-2020 #3 search: (TS = (“candida” OR “candida/albicans” OR “candida tropicalis” OR “candida glabrata” OR “candida albicans” OR “candida/analysis” OR “candida chemistry” OR “candida/drug effects” OR “candida infection” OR “candida albicans/analysis”)) *AND* DOCUMENT TYPES: (Article) Indexes = SCI-EXPANDED, SSCI, A&HCI, CPCI-S, CPCI-SSH, ESCI Tempo estipulado = 2015-2020 #1 AND #2 AND #3

Lilacs	#1 tw:((tw:(tw:((tw:(“chemical synthesis”)) OR (tw:(“Method, Organic Synthesis”)) OR (tw:(“Synthesis Technique, Organic”)) OR (tw:(“Synthesis Techniques, Organic”)) OR (tw:(“Technique, Organic Synthesis”)) OR (tw:(“Techniques, Organic Synthesis”)) OR (tw:(“Methods of Organic Synthesis”)) OR (tw:(“Organic Synthesis Methods”)) OR (tw:(“Methods, Organic Synthesis”)) OR (tw:(“Organic Synthesis”)) OR (tw:(“Syntheses, Organic”)) OR (tw:(“Synthesis, Organic”)))))) AND (fulltext:(“1”) AND db:(“LILACS”)) AND (year_cluster:[2015 TO 2020]) #2 tw:((tw:(tw:((tw:(“candida”)) OR (tw:(“candida/albicans”)) OR (tw:(“candida tropicalis”)) OR (tw:(“candida glabrata”)) OR (tw:(“candida albicans”)) OR (tw:(“candida/analysis”)) OR (tw:(“candida chemistry”)) OR (tw:(“candida/drug effects”)) OR (tw:(“candida infection”)) OR (tw:(“candida albicans/analysis”)))))) AND (fulltext:(“1”) AND db:(“LILACS”)) AND (year_cluster:[2015 TO 2020]) #3 tw:((tw:(tw:((tw:(“Agents,antifungal”)) OR (tw:(“antifungal agents”)) OR (tw:(“antifungal antibiotics”)) OR (tw:(“antibiotics,antifungal”)) OR (tw:(“antibiotics,antifungal”)) OR (tw:(“fungicides,therapeutic”)) OR (tw:(“therapeutic fungicides”)) OR (tw:(“fungicides industrial”)))))) AND (fulltext:(“1”) AND db:(“LILACS”)) AND (year_cluster:[2015 TO 2020]) #1 AND #2 AND #3

SciFinder	“agents”; “antifungal”; “chemical synthesis”; and “candida” Advanced search: Publication years: 2015-2020 Document types: Journal Refine: Research topic: microdilution test

**Table 2:** Descriptive analysis of the MIC results (*μ*g/mL) of the selected studies.

	Group 1: all strains	Group 2: all ATCC strains	Group 3: *C. albicans* strains	Group 4: *C. albicans* ATCC strains
Minimum value	0.0078	0.0078	0.0078	0.0078
First quartile (25%)	1.95	2	1.95	3.515
Second quartile (50%)	16	25	16	25
Third quartile (75%)	100	100	64	100
Maximum value	2000	2000	2000	2000

**Table 3:** Established parameters based on the minimum inhibitory concentrations of molecules synthesized for anti-*Candida* activity.

MIC range	Intensity of antifungal activity	Score
<3.515 *μ*g/mL	Very strong bioactivity	+++++
3.516-25 *μ*g/mL	Strong bioactivity	++++
26-100 *μ*g/mL	Moderate bioactivity	+++
101-500 *μ*g/mL	Weak bioactivity	++
500-2000 *μ*g/mL	Very weak bioactivity	+
>2000	No bioactivity	—

## Supplementary Materials

Supplementary Materials Table 1S. Data extraction from the included studies.
