# Supplementary material for: Breakpoints for the Classification of Anti-Candida Compounds in Antifungal Screening
Source: Biomed Res Int. 2021 Apr 6;2021:6653311. doi: 10.1155/2021/6653311 (PMC8046529; doi:10.1155/2021/6653311)
Supplement: Supplementary Materials — Table 1S. Data extraction from the included studies. [file 6653311.f1.docx]

| **Table 1S. Data extraction of the included studies** | | | |
| --- | --- | --- | --- |
| **Author, year** | **Substances ( Number of new substances)** | ***Candida* species (Reference)** | **MIC values range (µg/mL)** |
| Abonia et al., 2018  [1] | Naftifine and Analogues (6) | *C. albicans* (ATCC 10231) | 15.6 - 250 |
| Acosta et al., 2015  [2] | Pyrazolo[3,4-g][1,8]naphthyridin-5-amine (9) | *C. albicans* (ATCC 10231) | 31.2 - 250 |
| Afsarian et al., 2019  [3] | Tetrazole Derivatives (15) | *C. albicans* (ATCC10231) | 128 - 512 |
|  |  | *C. glabrata* (ATCC) | 256 - 512 |
|  |  | *C. krusei* (ATCC) | 128 - 512 |
|  |  | *C. parapsilosis* (ATCC 22019) | 128 - 512 |
| Akolkar et al., 2019  [4] | N -phenylacetamide-incorporated 1,2,3-triazoles (21) | *C. albicans* (ATCC NCIM 3471) | 25 - 200 |
| AlFindee et al., 2018  [5] | Carbohydrate esters (5) | *C. albicans* (ATCC MYA 2876) | 64 - 256 |
| Ameri et al., 2018  [6] | Aldimine-Type Schiff Base Derivatives (6) | *C. albicans* (ATCC 10231) | 16 - 512 |
|  |  | *C. utilis* (ATCC) | 32 - 512 |
| Ameri et al., 2016  [7] | Aldimine-Type Schiff Bases of 4-Amino-5-[(3,4,5-trimethoxyphenyl)methyl]-1,2,4-triazole-3-thione/thiol (6) | *C albicans* (ATCC10231) | 16 - 256 |
|  |  | *C. utilis* (ATCC) | 16 - 256 |
|  |  | *C. neoformans* (ATCC) | 32 - 1024 |
| Ammar at al., 2020  [8] | thiadiazino and thiazolo quinoxaline hybrids (20) | *C. albicans (ATCC10231)* | 3.9 - 166.6 |
| An et al., 2016  [9] | Series of 1,5-Benzodiazepine Derivatives (2) | *C. neoformans* (ATCC 32264) | 30 - 35 |
| Andrade et al., 2018  [10] | chalcone derivatives (8) | *C. albicans* (ATCC 10231) | 15.6 - 1000 |
|  |  | *C. glabrata* (ATCC) | 15.6 - 1000 |
|  |  | *C. krusei* (ATCC 34155) | 15.6 - 1000 |
|  |  | *C. tropicalis* (ATCC 28707) | 15.6 - 31.25 |
| Küçük et al., 2019  [11] | 1,2,3-thiadizoles and 1,2,3-selenadiazoles (6) | *C. albicans* (ATCC 10231) | 19.53 - 78.12 |
|  |  | *C. parapsilosis* (ATCC 22019) | 9.76 - 312.15 |
|  |  | *C. tropicalis* (ATCC 750) | 2.44 - 312.15 |
| Caneschi et al., 2017  [12] | Derived from amino alcohols (12) | *C. albicans* (ATCC 10231) | 15.65 - 625 |
| Cao et al., 2015  [13] | thienopyrrolidone derivatives (13) | *C. albicans* (ATCC SC 5314) | 0.0078 - 0.03125 |
|  |  | *C. albicans* (ATCC Y0109) | 0.0156 - 0.5 |
|  |  | *C. parapsilosis* (ATCC 22019) | 0.0156 - 0.5 |
|  |  | *C. glabrata* (ATCC 537) | 0.0156 - 4 |
| Chander et al., 2016  [14] | tetrahydroquinoline carbamates (11) | *C. albicans* (ATCC MTCC 3958) | 8 - 128 |
| Chate et al., 2019  [15] | Coumarin-pyrazolines (18) | *C. albicans (*ATCC NCIM 3471) | 20 - 69 |
|  |  | *C. glabrata* (ATCC 36909 NCYC 388) | 20 - 62 |
| Chudzik et al., 2019  [16] | 1,3,4-thiadiazole derivative (1) | *C. albicans* (NCPF 3153) | 96 |
|  |  | *C. parapsilosis* (ATCC 22019) | 64 |
| Araújo Neto et al., 2017  [17] | 5-nitro-thiophene-thiosemicarbazones derivatives (9) | *C. parapsilosis* (ATCC 22019) | 8 |
|  |  | *C. albicans* (ATCC 14053) | 1 - 16 |
|  |  | *C. krusei* (ATCC 6258) | 8 - 16 |
| Cruz-Claure et al., 2019  [18] | Symmetrical selenoesters (9) | *C. glabrata* (ATCC 2001) | 2 - 64 |
|  |  | *C. krusei* (ATCC 6258) | 1 - 64 |
|  |  | *C. parapsilosis* (ATCC 22019) | 0.5 - 64 |
| de Sá et al., 2018  [19] | Bis-arylidenecyclo-alkanone (1) | *C. albicans* (ATCC SC 5314) | 16 |
|  |  | *C. albicans* (ATCC 18804) | 4 |
|  |  |  |  |
|  |  |  |  |
| Desai et al., 2016  [20] | fluorinated pyrazole encompassing pyridyl 1,3,4-oxadiazole motifs(16) | *C. albicans* (ATCC MTCC 227) | 25 - 500 |
| Doğan et al., 2017  [21] | azole derivatives (32) | *C. albicans* (ATCC 90028) | 0.125 - 256 |
|  |  | *C. krusei* (ATCC 6258) | 4 - 256 |
|  |  | *C. parapsilosis* (ATCC 90018) | 0.125 - 256 |
| Elias et al., 2019  [22] | Coumarin-based antifungal azoles(21) | *C. albicans* (ATCC 90028) | 0.015 - 64 |
|  |  | *C. albicans* (NCBI P-87) | 0.015 - 64 |
|  |  | *C. glabrata* (ATCC 66032) | 1 - 64 |
|  |  | *C. glabrata* (ATCC 2001) | 2 - 64 |
|  |  | *C. glabrata* (NCBI 192) | 0.25 - 16 |
|  |  | *C. parapsilosis* (ATCC 90018) | 0.015 - 2 |
|  |  | *C. parapsilosis* (ATCC 22019) | 0.06 - 8 |
| Fabbro et al., 2016  [23] | Silver molybdate microcrystals (3) | Candida albicans (ATCC 90028) | 7.81 - 15.62 |
| Fesenko et al., 2019  [24] | 4-(tosylmethyl)semicarbazones (4) | *Candida albicans* (ATCC 90028) | 16 |
| Fonseca et al., 2019  [25] | Cobalt(II) Complexes with Ligands Derived from Pyrazoles and Dinitrobenzoate (13) | *Candida albicans* (ATCC 10231) | 125 - 2000 |
|  |  | *Candida tropicalis* (ATCC 20366) | 125 - 2000 |
|  |  | *Candida krusei* (ATCC 14243) | 31.25 - 2000 |
| Fosso et al., 2018  [26] | Amphiphilic Tobramycin (6) | *Candida albicans* (ATCC 10231) | 3.9 - 62.5 |
|  |  | *Candida albicans* (ATCC MYA-2876) | 15.6 - 62.5 |
|  |  | *Candida albicans* (ATCC MYA-2310) | 7.8 - 62.5 |
|  |  | *Candida glabrata* (ATCC 2001) | 7.8 - 62.5 |
|  |  | *Candida krusei* (ATCC 6258) | 3.9 - 31.3 |
|  |  | *Candida parapsilosis* (ATCC 22019) | 1.95 - 31.3 |
| Gill et al., 2015  [27] | Synthetic anti-fungal tripeptide (5) | *Candida krusei* (ATCC 6258) | 171.25 - 382 |
| Giurg et al., 2017  [28] | bis[(2-chlorocarbonyl)phenyl] (26) | *Candida albicans* (ATCC 10231) | 16 - 62 |
|  |  | *Candida glabrata* (ATCC 90030) | 16 - 64 |
| Gondru et al, 2018  [29] | Pyrazole-Thiazole Hybrids (4) | *Candida albicans* (ATCC MTCC 3017) | 7.8 |
|  |  | *Candida albicans* (ATCC MTCC 227) | 3.9 |
|  |  | *Candida albicans* (ATCC MTCC 1637) | 3.9 |
| Gonzaléz et al., 2016  [30] | 1′-homo-N-1,2,3-triazol-bicyclic carbonucleosides (2) | *Candida albicans* (ATCC-10231) | 0.25 - 2 |
|  |  | *Candida tropicalis* (ATCC 13803) | 0.5 - 2 |
|  |  | *Candida utilis* (ATCC 9226) | 0.12 |
| Gonzaléz et al., 2016  [31] | Azide-enolate 1,3-dipolar cycloaddition (7) | *Candida utilis* (ATCC 9226) | 0.25 - 16 |
|  |  | *Candida albicans* (ATCC 10231) | 0.03 - 16 |
|  |  | *Candida tropicalis* (ATCC 13803) | 0.03 - 16 |
| Hashemi et al., 2015  [32] | Fluconazole analogs with triazole-modified scaffold (18) | *C. albicans* (ATCC18804) | 0.25 - 64 |
|  |  | *C. albicans* (PTCC 6027) | 0.03 - 64 |
| Ji et al., 2019  [33] | 5-(piperazin-1-yl)quinolin-2(1H)-one derivatives (33) | *C. albicans* (ATCC 76615) | 32 - 128 |
|  |  | *C. neoformans* (ATCC 32719) | 128 - 512 |
| Ji et al.,2016  [34] | phosphoramidate derivatives of coumarin (20) | *Candida albicans* (CMCC 76615) | 8 - 128 |
| Jia et al., 2018  [35] | Jelleine- (1) | *Candida glabrata* | 30 |
|  |  | Candida al*bicans* | 61 |
|  |  | *Candida tropicalis* | 15 |
|  |  | *Candida krusei* | 30 |
|  |  | *Candida parapsilosis* | 61 |
| Jóźwiak et al.,2018  [36] | Connections of Thiosemicarbazide, 1,2,4-Triazole and 1,3,4-Thiadiazole with Palmitic Acid (30) | *Candida albicans* (ATCC 10231) | 1.56 - 100 |
|  |  | *Candida albicans* (ATCC 30028) | 1.56 - 100 |
| Khan et al.,2019  [37] | N-(3-(-2-(7-Chloroquinolin-2-yl)vinyl) benzylidene)anilines (10) | *Candida albicans* (ATCC NCIM-3471) | 94.2 - 164.1 |
| Lazic, et al., 2018  [38] | Bis-guanylhydrazones (4) | *Candida albicans* (ATCC 10231) | 4 - 6.25 |
|  |  | *Candida parapsilosis* (ATCC 22019) | 2 - 6.2 |
|  |  | *C. glabrata* (ATCC2001) | 3.13 - 15.62 |
| Lone et al., 2018  [39] | 2-(((2-ether)amino)methylene)-dimedone derivatives (20) | *C. albicans* (ATCC 24433) | 32 - 256 |
|  |  | *C. albicans* (ATCC 90028) | 64 - 256 |
|  |  | *C. tropicalis* (ATCC 750) | 16 - 256 |
|  |  | *C. krusei (ATCC 6258)* | 16 - 256 |
|  |  | *C. utilis*  (ATCC 9950) | 32 - 128 |
| Lone et al., 2020  [40] | Eugenol Tosylate Congeners (7) | *C. albicans* (ATCC SC 5314) | 0.125 - 500 |
| Louvis et al., 2016  [41] | 3-aryl-1,4-naphthoquinones (4) | *C. albicans*  (ATCC 24433) | 0.8 - 16 |
|  |  | *C. krusei* (ATCC 34135) | 128 - 256 |
|  |  | *C. tropicalis* (ATCC 750) | 32 |
|  |  | *C. glabrata* (ATCC 90030) | 16 - 32 |
| Lum et al., 2015  [42] | Synthetic peptides (7) | *C. albicans* (ATCC SC 5314) | 8 - 64 |
|  |  | *C. albicans* (ATCC 90028) | 32 - 128 |
|  |  | *C. krusei* (ATCC 6258 ) | 16 - 64 |
|  |  | *C. parapsilosis* (ATCC 22019) | 8 - 128 |
| Madanchi et al., 2011  [43] | heptapeptide derived from Aurein1.2 (1) | *Candida albicans* (ATCC 10231) | 16 - 125 |
|  |  | *C. glabrata* (ATCC 90030) | 1.1 - 125 |
|  |  | *C. krusei* (ATCC 28870) | 32 - 500 |
| Montoya et al., 2016  [44] | Hybrid Molecules Containing a 7-Chloro-4-aminoquinoline Nucleus and a Substituted 2-Pyrazoline (24) | *C. albicans* (ATCC 10231) | 62.5 - 250 |
| Nguyen et al., 2015  [45] | Head-to-head and head-to-tail bisamidine compounds (43) | *C. glabrata* (ATCC 90030) | 0.06 - 32 |
|  |  | *C. parapsilosis* (ATCC 90028) | 0.1 - 8 |
| Niewiadomy et al., 2015  [46] | N-N-cyclic-2,4-dihydroxythiobenzamide derivatives (6) | *C. albicans* (ATCC 10231) | 100 - 200 |
| Ozdemir, et al., 2017  [47] | Pyrrole-Based chalcones (10) | *C. albicans* (ATCC 90028) | 50 - 200 |
|  |  | *C. glabrata* (ATCC 90030) | 100 - 200 |
|  |  | *C. parapsilosis* (ATCC 22019) | 50 - 100 |
|  |  | *C. krusei* (ATCC 6258) | 25 - 200 |
| Patil et al., 2018  [48] | Pyperazine-sulphonamide linked schiff bases (11) | *C. albicans* (NCIM 3471) | 39.6 - 247.8 |
| Ptaszynska et al., 2019  [49] | Antibiotic-based conjugates containing antirobial HLopt2 peptide (4) | *C. krusei* (DSM 6128 ATCC 6258) | 125 - 250 |
| Ptaszynska et al., 2019  [50] | Peptides conjugates of lactoferricin analogues and antirobials (8) | *C. albicans*  (ATCC 10231) | 250 |
|  |  | *C. krusei* (DSM 6128 - ATCC 6258) | 12.5 - 100 |
|  |  | *C. glabrata (DSM 11226 - ATCC 90030)* | 25 - 100 |
|  |  | *C. parapsilosis* (DSM 5784 - ATCC 22019) | 50 - 250 |
| Pulya et al., 2016  [51] | 4-(1-phenyl-1-hydroxyethyl)-1-(*o*-hydroxyphenyl)-1*H-*1,2,3-triazole (35) | *C. albicans* (ATCC 24433) | 4 - 128 |
|  |  | *C. albicans* (ATCC 10231) | 8 - 128 |
|  |  | *C. glabrata*  (NCYC 388 - ATCC 36909) | 8 - 128 |
| Ramirez-Vilalva et al., 2017  [52] | Oxazolidin-2-one linked -1,2,3-triazole derivatives (11) | *C. albicans* (ATCC 10231) | 1 - 8 |
|  |  | *C. tropicalis* (ATCC 13803) | 8 |
|  |  | *C. utilis* (ATCC 9226) | 4 - 8 |
|  |  | *C. parapsilosis* (ATCC 22019) | 0.25 - 8 |
|  |  | *C. glabrata* (ATCC 34138) | 0.12 - 4 |
|  |  | *C. krusei* (ATCC 14243) | 0.25 - 8 |
| Ramirez-Villalva et al., 2015  [53] | miconazole analogues (4) | *C. albicans* (ATCC 10231) | 0.06 - 0.12 |
|  |  | *C. utilis* (ATCC 9226) | 0.12 - 1 |
|  |  | *C. tropicalis* (ATCC 13803) | 0.25 - 1 |
| Ravichandran et al., 2018  [54] | Sodium alginate-amphotericin B conjugates (12) | *C. albicans* (ATCC - NCIM 3102) | 0.195 - 1.562 |
|  |  | *C. parapsilosis* (ATCC 7330) | 0.048 - 1.562 |
| Sardi et al., 2016  [55] | Caffeic acid derivative esters (8) | *Candida albicans* (ATCC 90028) | 7.81 - 31.25 |
| Sharma et al., 2020  [56] | 3-amidocoumarins  (23) | *Candida albicans* (ATCC - MTCC 3017) | 6.25 - 200 |
| Shrestha et al., 2017  [57] | Dimeric  cationic triazolium  (6) | *Candida albicans* (ATCC-MYA2876) | 3.91 |
| Shrestha, 2017  [58] | Alkylated azoles (28) | *Candida albicans* (ATCC 10231) | 0.24 - 15.6 |
|  |  | *Candida albicans* (ATCC- MYA 2876) | 0.975 - 15.6 |
|  |  | *Candida albicans* (ATCC MYA-2310) | 0.975 - 31.3 |
|  |  | *Candida glabrata* (ATCC 2001) | 0.975 - 31.3 |
|  |  | *Candida krusei* (ATCC 62580) | 0.06 - 7.8 |
|  |  | *Candida parapsilosis* (ATCC 22019) | 0.06 - 3.9 |
| Singh et al.,2015  [59] | 4-methyl-7-O-substituted coumarins(9) | *Candida albicans* (ATCC MTCC 1346) | 6.25 - 25 |
| Stiz et al., 2016  [60] | Cyclic Imides (5) | *Candida tropicalis* (PMC 0912) | 32 - 64 |
|  |  | *Candida krusei* (DSM6128 - ATCC 6258) | 4 - 32 |
|  |  | *Candida parapsilosis* (DSM 11224 - ATCC 90030) | 8 - 32 |
|  |  | *Candida parapsilosis* (ATCC 22019) | 4 - 16 |
|  |  | *Candida albicans* (ATCC 10231) | 8 - 64 |
|  |  | *Candida albicans* (ATCC 24433) | 4 - 64 |
|  |  | *Candida albicans* (ATCC 20891) | 4 - 64 |
|  |  | *Candida albicans (*ATCC 28367) | 16 - 64 |
| Subedi et al., 2018  [61] | azole and quinone hybridized phosphonates (11) | *Candida albicans* | 16 - 128 |
| Subhedar et al., 2018  [62] | Quinolidinyl-2,4-  thiazolidinones  (14) | *Candida albicans (*ATCC 24433) | 32 - 256 |
|  |  | *Candida albicans (*ATCC 10231) | 16 - 128 |
|  |  | *Candida glabrata* (ATCC - NCYC 488) | 32 - 128 |
| Terra et al.,2018  [63] | 1,3-Benzoxathiol-2-one Derivatives (2) | *Candida albicans* (ATCC 24433) | 16 - 32 |
|  |  | *Candida krusei* (ATCC 34135) | 4 - 16 |
|  |  |  |  |
|  |  | *Candida parapsilosis* (ATCC 90018) | 32 - 64 |
|  |  | *Candida glabrata* (ATCC 90030) | 16 - 32 |
|  |  | *Candida tropicalis* (ATCC 750) | 16 - 32 |
| Tevyashova et al., 2016  [64] | Conjugates of amphotericin B(12) | *Candida albicans (*ATCC 14053) | 0.5 - 8 |
| Thanh et al., 2015  [65] | Thiosemicarbazones of Substituted  Benzaldehydes and N-(Hepta-O-Acetyl-β-d-  Lactosyl)Thiosemicarbazide  (19) | *Candida albicans* (ATCC 7754) | 0.25 - 0.58 |
| Tiwari et al., 2017  [66] | Coumarin Derivatives (15) | *Candida albicans* (ATCC- NCIM 3471) | 25 - 66 |
|  |  | *Candida glabrata* (ATCC- 36909 NCYC 388) | 26 - 64 |
| Ünver et al., 2019  [67] | Hydrazone Derivatives(5) | *Candida albicans* (ATCC 22019) | 125 - 250 |
| Vasile et al., 2018  [68] | Carboxylate Complexes (4) | *Candida albicans* (ATCC 10231) | 128 |
| Victor et al., 2019  [69] | 1,4-Disubstituted-1,2,3-bistriazoles (2) | *Candida krusei* (ATCC 6258) | 32 |
|  |  | *Candida tropicalis* (ATCC 750) | 64 |
| Whang et al., 2018  [70] | 4-chloro-2H-thiochromenes (34) | *Candida albicans* (CPCC 400616) | 0.5 - 64 |
|  |  | *Candida albicans* (ATCC SC5314) | 0.5 - 64 |
|  |  | *Candida tropicalis* (ATCC - CGMCC 23739) | 2 - 128 |
| Wang et al.,2018  [71] | Purine benzimidazoles (27) | *Candida albicans* (ATCC 90023) | 32 - 256 |
|  |  | *Candida parapsilosis* (ATCC 22019) | 32 - 512 |
| Yurttas et al., 2016  [72] | Benzothiazole derivatives (19) | *Candida parapsilosis* (ATCC 22109) | 100 - 200 |
|  |  | *Candida glabrata* (ATCC 66032) | 100 - 200 |
|  |  | *Candida albicans* (ATCC 24433) | 100 - 200 |
|  |  | *Candida krusei* (ATCC 6258) | 100 - 200 |
|  |  | *Candida tropicalis* (ATCC 750) | 100 - 200 |
| Zhang et al., 2019  [73] | Amphotericin  B derivatives  (9) | *Candida albicans* (ATCC10231) | 2 - 16 |
|  |  | *Candida albicans* (ATCC 90028) | 2 - 16 |
| Zhang et al., 2018  [74] | Carbazole-triazole conjugates (11) | *Candida albicans* (ATCC 90023) | 8 - 128 |
|  |  | *Candida parapsilosis* (ATCC 22019) | 2 - 64 |
| Zhao et al., 2017  [75] | Biphenyl imidazole derivatives (27) | *Candida albicans* (ATCC SC5314) | 0.03125 - 16 |
|  |  | *Candida albicans* (CPCC 400523) | 0.125 - 8 |
|  |  | *Candida tropicalis* (ATCC - CGMCC 23739) | 0.03125 - 8 |
| Zhao et al., 2019  [76] | 𝛽-azolephenylacetone  derivatives  (32) | *Candida albicans* (ATCC- SC 5314) | 0.025 - 8 |
| Zhao et al., 2019  [77] | L-amino alcohol derivatives (32) | *Candida albicans* (CPCC 400523) | 0.04 - 4 |
|  |  | *Candida albicans* (ATCC SC5314) | 0.06 - 0.5 |
|  |  | *Candida tropicalis* (ATCC-CGMCC 23739) | 0.06 - 8 |
| Zhao et al.,2018  [78] | Benzoheterocycle analogues (27) | *Candida albicans* (ATCC SC5314) | 0.125 - 64 |
|  |  | *Candida albicans* (CPCC 400523) | 0.25 - 64 |
|  |  | *Candida tropicalis* (ATCC-CGMCC 23739) | 0.125 - 64 |
| Zhao et al., 2016  [79] | Benzothiazole derivatives (25) | *Candida albicans* (ATCC SC5314) | 0.125 - 8 |
|  |  | *Candida albicans* (CPCC 400523) | 0.25 - 64 |
|  |  | *Candida tropicalis* (ATCC -CGMCC 23739) | 0.125 - 16 |

REFERENCES

1. R. Abonia, A. Garay, J. C. Castillo, B. Insuasty, J. Quiroga, J. C. Castillo, M. Nogueras, J. Cobo, E. Butassi and S. Zacchino, "Design of Two Alternative Routes for the Synthesis of Naftifine and Analogues as Potential Antifungal Agents," *Molecules (Basel, Switzerland)*, vol. 23, no. 3, 2018.

2. P. Acosta, E. Butassi, B. Insuasty, A. Ortiz, R. Abonia, S. A. Zacchino and J. Quiroga, "Microwave-assisted synthesis of novel pyrazolo[3,4-g][1,8]naphthyridin-5-amine with potential antifungal and antitumor activity," *Molecules*, vol. 20, no. 5, pp. 8499-8520, 2015.

3. M. H. Afsarian, M. Farjam, E. Zarenezhad, S. Behrouz and M. N. S. Rad, "Synthesis, antifungal evaluation and molecular docking studies of some tetrazole derivatives," *Acta Chimica Slovenica*, vol. 66, no. 4, pp. 874-887, 2019.

4. S. V. Akolkar, A. A. Nagargoje, V. S. Krishna, D. Sriram, J. N. Sangshetti, M. Damale and B. B. Shingate, "New: N -phenylacetamide-incorporated 1,2,3-triazoles: [Et<inf>3</inf>NH][OAc]-mediated efficient synthesis and biological evaluation," *RSC Advances*, vol. 9, no. 38, pp. 22080-22091, 2019.

5. M. N. AlFindee, Q. Zhang, Y. P. Subedi, J. P. Shrestha, Y. Kawasaki, M. Grilley, J. Y. Takemoto and C.-W. T. Chang, "One-step synthesis of carbohydrate esters as antibacterial and antifungal agents," *Bioorganic & medicinal chemistry*, vol. 26, no. 3, pp. 765-774, 2018.

6. A. Ameri, G. Khodarahmi, H. Forootanfar, F. Hassanzadeh and G.-H. Hakimelahi, "Hybrid Pharmacophore Design, Molecular Docking, Synthesis, and Biological Evaluation of Novel Aldimine-Type Schiff Base Derivatives as Tubulin Polymerization Inhibitor," *Chemistry & biodiversity*, vol. 15, no. 3, pp. e1700518-e1700518, 2018.

7. A. Ameri, G. Khodarahmi, F. Hassanzadeh, H. Forootanfar and G.-H. Hakimelahi, "Novel Aldimine-Type Schiff Bases of 4-Amino-5-[(3,4,5-trimethoxyphenyl)methyl]-1,2,4-triazole-3-thione/thiol: Docking Study, Synthesis, Biological Evaluation, and Anti-Tubulin Activity," *Archiv der Pharmazie*, vol. 349, no. 8, pp. 662-681, 2016.

8. Y. A. Ammar, A. A. Farag, A. M. Ali, S. A. Hessein, A. A. Askar, E. A. Fayed, D. M. Elsisi and A. Ragab, "Antimicrobial evaluation of thiadiazino and thiazolo quinoxaline hybrids as potential DNA gyrase inhibitors; design, synthesis, characterization and morphological studies," *Bioorganic Chemistry*, vol. 99, 2020.

9. Y.-S. An, Z.-F. Hao, X.-J. Zhang and L.-Z. Wang, "Efficient Synthesis and Biological Evaluation of a Novel Series of 1,5-Benzodiazepine Derivatives as Potential Antimicrobial Agents," *Chemical biology & drug design*, vol. 88, no. 1, pp. 110-121, 2016.

10. J. T. Andrade, F. R. S. Santos, W. G. Lima, C. D. F. Sousa, L. S. F. M. Oliveira, R. I. M. A. Ribeiro, A. J. P. S. Gomes, M. G. F. Araújo, J. A. F. P. Villar and J. M. S. Ferreira, "Design, synthesis, biological activity and structure-activity relationship studies of chalcone derivatives as potential anti-Candida agents," *The Journal of antibiotics*, vol. 71, no. 8, pp. 702-712, 2018.

11. H. Başpınar Küçük, Z. Banu Salt, E. Mataracı Kara, A. Sayık Mehan and A. S. Yusufoğlu, "Synthesis of novel 1,2,3-thiadizoles and 1,2,3-selenadiazoles as new antimicrobial agents," *Phosphorus, Sulfur and Silicon and the Related Elements*, vol. 194, no. 9, pp. 903-908, 2019.

12. C. A. Caneschi, A. M. d. Almeida, F. J. Martins, M. L. Hyaric, M. M. E. Oliveira, G. C. Macedo, M. V. d. Almeida and N. R. B. Raposo, "In vitro antifungal activity of organic compounds derived from amino alcohols against onychomycosis," *Brazilian journal of microbiology : [publication of the Brazilian Society for Microbiology]*, vol. 48, no. 3, pp. 476-482, 2017.

13. X. Cao, Y. Xu, W. Chu, Y. Cao, R. Wang, R. Zhou and Y. Yang, "Design, synthesis, and structure-activity relationship studies of novel thienopyrrolidone derivatives with strong antifungal activity against Aspergillus fumigates," *European journal of medicinal chemistry*, vol. 102, pp. 471-476, 2015.

14. S. Chander, P. Ashok, Y.-T. Zheng, P. Wang, K. S. Raja, A. Taneja and S. Murugesan, "Design, synthesis and in-vitro evaluation of novel tetrahydroquinoline carbamates as HIV-1 RT inhibitor and their antifungal activity," *Bioorganic chemistry*, vol. 64, pp. 66-73, 2016.

15. A. V. Chate, A. A. Redlawar, G. M. Bondle, A. P. Sarkate, S. V. Tiwari and D. K. Lokwani, "A new efficient domino approach for the synthesis of coumarin-pyrazolines as antimicrobial agents targeting bacterial d-alanine-d-alanine ligase," *New Journal of Chemistry*, vol. 43, no. 23, pp. 9002-9011, 2019.

16. B. Chudzik, K. Bonio, W. Dabrowski, D. Pietrzak, A. Niewiadomy, A. Olender, B. Pawlikowska-Pawlęga and M. Gagoś, "Antifungal effects of a 1,3,4-thiadiazole derivative determined by cytochemical and vibrational spectroscopic studies," *PloS one*, vol. 14, no. 9, pp. e0222775-e0222775, 2019.

17. L. N. de Araújo Neto, M. d. C. A. de Lima, J. F. de Oliveira, E. R. de Souza, M. D. S. Buonafina, M. N. V. Anjos, F. A. Brayner, L. C. Alves, R. P. Neves and F. J. B. Mendonça-Junior, "Synthesis, cytotoxicity and antifungal activity of 5-nitro-thiophene-thiosemicarbazones derivatives," *Chemico-Biological Interactions*, vol. 272, pp. 172-181, 2017.

18. M. L. De la Cruz-Claure, A. A. Cèspedes-Llave, M. T. Ulloa, M. Benito-Lama, E. Domínguez-álvarez and A. Bastida, "Inhibition–disruption of Candida glabrata biofilms: Symmetrical selenoesters as potential anti-biofilm agents," *Microorganisms*, vol. 7, no. 12, 2019.

19. N. P. de Sá, L. F. J. de Paula, L. F. F. Lopes, L. I. B. Cruz, T. T. S. Matos, C. I. Lino, R. B. de Oliveira, E. M. de Souza-Fagundes, B. B. Fuchs, E. Mylonakis and S. Johann, "In vivo and in vitro activity of a bis-arylidenecyclo-alkanone against fluconazole-susceptible and -resistant isolates of Candida albicans," *Journal of global antimicrobial resistance*, vol. 14, pp. 287-293, 2018.

20. N. C. Desai, G. M. Kotadiya, A. R. Trivedi, V. M. Khedkar and P. C. Jha, "Design, synthesis, and biological evaluation of novel fluorinated pyrazole encompassing pyridyl 1,3,4-oxadiazole motifs," *Medicinal Chemistry Research*, vol. 25, no. 11, pp. 2698-2717, 2016.

21. I. S. Dogan, S. Sari, S. Dalkara, S. Sarac, D. Kart, G. S. Essiz and I. Vural, "New azole derivatives showing antimicrobial effects and their mechanism of antifungal activity by molecular modeling studies," *European journal of medicinal chemistry*, vol. 130, pp. 124-138, 2017.

22. R. Elias, R. I. Benhamou, Q. Z. Jaber, O. Dorot, S. L. Zada, K. Oved, E. Pichinuk and M. Fridman, "Antifungal activity, mode of action variability, and subcellular distribution of coumarin-based antifungal azoles," *European journal of medicinal chemistry*, vol. 179, pp. 779-790, 2019.

23. M. T. Fabbro, C. C. Foggi, L. P. S. Santos, L. Gracia, A. Perrin, C. Perrin, C. E. Vergani, A. L. Machado, J. Andrés, E. Cordoncillo and E. Longo, "Synthesis, antifungal evaluation and optical properties of silver molybdate microcrystals in different solvents: a combined experimental and theoretical study," *Dalton transactions (Cambridge, England : 2003)*, vol. 45, no. 26, pp. 10736-10743, 2016.

24. A. A. Fesenko, A. N. Yankov and A. D. Shutalev, "A general and convenient synthesis of 4-(tosylmethyl)semicarbazones and their use in amidoalkylation of hydrogen, heteroatom, and carbon nucleophiles," *Tetrahedron*, vol. 75, no. 45, 2019.

25. D. Fonseca, M. A. Macias, J. J. Hurtado, S. M. Leal-Pinto, M. V. Roa-Cordero, J. D. Vargas, E. M. Moreno-Moreno, L. Suescun and A. Munoz-Castro, "Inhibition of C. albicans Dimorphic Switch by Cobalt(II) Complexes with Ligands Derived from Pyrazoles and Dinitrobenzoate: Synthesis, Characterization and Biological Activity," *International journal of molecular sciences*, vol. 20, no. 13, 2019.

26. M. Y. Fosso, S. K. Shrestha, N. Thamban Chandrika, E. K. Dennis, K. D. Green and S. Garneau-Tsodikova, "Differential Effects of Linkers on the Activity of Amphiphilic Tobramycin Antifungals," *Molecules (Basel, Switzerland)*, vol. 23, no. 4, 2018.

27. K. Gill, S. Kumar, I. Xess and S. Dey, "Novel synthetic anti-fungal tripeptide effective against Candida krusei," *Indian journal of medical microbiology*, vol. 33, no. 1, pp. 110-116, 2015.

28. M. Giurg, A. Gołąb, J. Suchodolski, R. Kaleta, A. Krasowska, E. Piasecki and M. Piętka-Ottlik, "Reaction of bis[(2-chlorocarbonyl)phenyl] Diselenide with Phenols, Aminophenols, and Other Amines towards Diphenyl Diselenides with Antimicrobial and Antiviral Properties," *Molecules (Basel, Switzerland)*, vol. 22, no. 6, 2017.

29. R. Gondru, K. Sirisha, S. Raj, S. K. Gunda, C. G. Kumar, M. Pasupuleti and R. Bavantula, "Design, Synthesis, In Vitro Evaluation and Docking Studies of Pyrazole-Thiazole Hybrids as Antimicrobial and Antibiofilm Agents," *ChemistrySelect*, vol. 3, no. 28, pp. 8270-8276, 2018.

30. D. González-Calderón, M. G. Mejía-Dionicio, M. A. Morales-Reza, J. G. Aguirre-de Paz, A. Ramírez-Villalva, M. Morales-Rodríguez, A. Fuentes-Benítes and C. González-Romero, "Antifungal activity of 1′-homo-N-1,2,3-triazol-bicyclic carbonucleosides: A novel type of compound afforded by azide-enolate (3+2) cycloaddition," *Bioorganic Chemistry*, vol. 69, pp. 1-6, 2016.

31. D. González-Calderón, M. G. Mejía-Dionicio, M. A. Morales-Reza, A. Ramírez-Villalva, M. Morales-Rodríguez, B. Jauregui-Rodríguez, E. Díaz-Torres, C. González-Romero and A. Fuentes-Benítes, "Azide-enolate 1,3-dipolar cycloaddition in the synthesis of novel triazole-based miconazole analogues as promising antifungal agents," *European journal of medicinal chemistry*, vol. 112, pp. 60-65, 2016.

32. S. M. Hashemi, H. Badali, H. Irannejad, M. Shokrzadeh and S. Emami, "Synthesis and biological evaluation of fluconazole analogs with triazole-modified scaffold as potent antifungal agents," *Bioorganic & medicinal chemistry*, vol. 23, no. 7, pp. 1481-1491, 2015.

33. Q. Ji, Q. Deng, B. Li, B. Li and Y. Shen, "Design, synthesis and biological evaluation of novel 5-(piperazin-1-yl)quinolin-2(1H)-one derivatives as potential chitin synthase inhibitors and antifungal agents," *European journal of medicinal chemistry*, vol. 180, pp. 204-212, 2019.

34. Q. Ji, Z. Ge, K. Chen, H. Wu, X. Liu, Y. Huang, L. Yuan, Z. Ge, X. Yang and F. Liao, "Synthesis and biological evaluation of novel phosphoramidate derivatives of coumarin as chitin synthase inhibitors and antifungal agents," *European journal of medicinal chemistry*, vol. 108, pp. 166-176, 2016.

35. F. Jia, J. Wang, J. Peng, P. Zhao, Z. Kong, K. Wang, W. Yan and R. Wang, "The in vitro, in vivo antifungal activity and the action mode of Jelleine-I against Candida species," *Amino acids*, vol. 50, no. 2, pp. 229-239, 2018.

36. M. Jozwiak, M. Wrzosek, W. Olejarz, G. Kubiak-Tomaszewska, M. Jozwiak, M. Jozwiak, K. Stepien, M. Wrzosek, W. Olejarz, M. Struga, K. Stepien, A. Filipowska, W. Filipowski and M. Struga, "Synthesis, Structural Studies and Biological Evaluation of Connections of Thiosemicarbazide, 1,2,4-Triazole and 1,3,4-Thiadiazole with Palmitic Acid," *Molecules (Basel, Switzerland)*, vol. 23, no. 4, 2018.

37. F. A. K. Khan, R. N. Kaduskar, R. Patil, R. H. Patil, S. A. Ansari, H. M. Alkahtani, A. A. Almehizia, D. B. Shinde and J. N. Sangshetti, "Synthesis, biological evaluations and computational studies of N-(3-(-2-(7-Chloroquinolin-2-yl)vinyl) benzylidene)anilines as fungal biofilm inhibitors," *Bioorganic & medicinal chemistry letters*, vol. 29, no. 4, pp. 623-630, 2019.

38. J. Lazic, V. Ajdacic, M. Zlatovic, I. Opsenica, J. Lazic, S. Vojnovic, J. Nikodinovic-Runic, M. Pekmezovic and S. Mogavero, "Bis-guanylhydrazones as efficient anti-Candida compounds through DNA interaction," *Applied microbiology and biotechnology*, vol. 102, no. 4, pp. 1889-1901, 2018.

39. A. M. Lone, M. A. Rather, M. A. Bhat, Z. S. Bhat, I. Q. Tantry and P. Prakash, "Synthesis and in vitro evaluation of 2-(((2-ether)amino)methylene)-dimedone derivatives as potential antimicrobial agents," *Microbial Pathogenesis*, vol. 114, pp. 431-435, 2018.

40. S. A. Lone, M. Y. Wani, P. Fru and A. Ahmad, "Cellular apoptosis and necrosis as therapeutic targets for novel Eugenol Tosylate Congeners against Candida albicans," *Scientific Reports*, vol. 10, no. 1, 2020.

41. A. D. R. Louvis, N. A. A. Silva, F. S. Semaan, F. D. C. Da Silva, G. Saramago, L. C. S. V. De Souza, B. L. A. Ferreira, H. C. Castro, J. P. Salles, A. L. A. Souza, V. F. Ferreira and D. D. L. Martins, "Synthesis, characterization and biological activities of 3-aryl-1,4-naphthoquinones-green palladium-catalysed Suzuki cross coupling," *New Journal of Chemistry*, vol. 40, no. 9, pp. 7643-7656, 2016.

42. K. Y. Lum, S. T. Tay, C. F. Le, V. S. Lee, N. H. Sabri, R. D. Velayuthan, H. Hassan and S. D. Sekaran, "Activity of Novel Synthetic Peptides against Candida albicans," *Scientific reports*, vol. 5, pp. 9657-9657, 2015.

43. H. Madanchi, A. A. Shabani, H. Madanchi, S. Sardari, V. Khalaj, S. Jang, K. R. Ebrahimi, M. S. J. Seyed and S. S. Kazemi, "AurH1: a new heptapeptide derived from Aurein1.2 antimicrobial peptide with specific and exclusive fungicidal activity," *Journal of peptide science : an official publication of the European Peptide Society*, vol. 25, no. 7, pp. e3175-e3175, 2019.

44. A. Montoya, J. Quiroga, R. Abonia, M. Derita, M. Sortino, A. Ornelas, S. Zacchino and B. Insuasty, "Hybrid Molecules Containing a 7-Chloro-4-aminoquinoline Nucleus and a Substituted 2-Pyrazoline with Antiproliferative and Antifungal Activity," *Molecules (Basel, Switzerland)*, vol. 21, no. 8, 2016.

45. S. T. Nguyen, S. M. Kwasny, X. Ding, J. D. Williams, N. P. Peet, T. L. Bowlin and T. J. Opperman, "Synthesis and antifungal evaluation of head-to-head and head-to-tail bisamidine compounds," *Bioorganic & medicinal chemistry*, vol. 23, no. 17, pp. 5789-5798, 2015.

46. A. Niewiadomy, A. Skrzypek, J. Matysiak, U. Głaszcz, J. Wietrzyk and E. Krajewska-Kułak, "SYNTHESIS AND BIOLOGICAL ACTIVITY OF NOVEL N,N-CYCLIC-2,4-DIHYDROXYTHIOBENZAMIDE DERIVATIVES," *Acta poloniae pharmaceutica*, vol. 72, no. 5, pp. 943-950, 2015.

47. A. Özdemir, M. D. Altıntop, B. Sever, H. K. Gençer, H. A. Kapkaç, Ö. Atlı and M. Baysal, "A New Series of Pyrrole-Based Chalcones: Synthesis and Evaluation of Antimicrobial Activity, Cytotoxicity, and Genotoxicity," *Molecules (Basel, Switzerland)*, vol. 22, no. 12, 2017.

48. R. H. Patil, K. F. A. Kalam, K. Jadhav, J. N. Sangshetti, M. Damale, A. S. Akber, H. M. Alkahtani, K. A. Ali, S. D. Shinde and R. Patil, "Fungal biofilm inhibition by piperazine-sulphonamide linked Schiff bases: Design, synthesis, and biological evaluation," *Archiv der Pharmazie*, vol. 351, no. 3-4, pp. e1700354-e1700354, 2018.

49. N. Ptaszyńska, K. Gucwa, K. Olkiewicz, A. Łȩgowska, J. Okońska, J. Ruczyński, A. Gitlin-Domagalska, D. Dȩbowski, S. S. Milewski, K. Rolka, N. Ptaszynska, K. Gucwa, K. Olkiewicz, L. g. Anna, J. Okonska, J. Ruczynski, A. Gitlin-Domagalska, D. b. Dawid, K. Rolka, K. Gucwa and S. S. Milewski, "Antibiotic-Based Conjugates Containing Antimicrobial HLopt2 Peptide: Design, Synthesis, Antimicrobial and Cytotoxic Activities," *ACS chemical biology*, vol. 14, no. 10, pp. 2233-2242, 2019.

50. N. Ptaszyńska, K. Olkiewicz, J. Okońska, K. Gucwa, A. Łęgowska, A. Gitlin-Domagalska, D. Dębowski, J. Lica, M. Heldt, S. Milewski, T. B. Ng and K. Rolka, "Peptide conjugates of lactoferricin analogues and antimicrobials—Design, chemical synthesis, and evaluation of antimicrobial activity and mammalian cytotoxicity," *Peptides*, vol. 117, 2019.

51. S. Pulya, Y. Kommagalla, D. G. Sant, S. U. Jorwekar, S. G. Tupe, M. V. Deshpande and C. V. Ramana, "Re-engineering of PIP3-antagonist triazole PITENIN's chemical scaffold: Development of novel antifungal leads," *RSC Advances*, vol. 6, no. 14, pp. 11691-11701, 2016.

52. A. Ramírez-Villalva, D. González-Calderón, R. I. Rojas-García, C. González-Romero, J. Tamaríz-Mascarúa, M. Morales-Rodríguez, N. Zavala-Segovia and A. Fuentes-Benítes, "Synthesis and antifungal activity of novel oxazolidin-2-one-linked 1,2,3-triazole derivatives," *MedChemComm*, vol. 8, no. 12, pp. 2258-2262, 2017.

53. A. Ramirez-Villalva, C. Gonzalez-Romero, D. Gonzalez-Calderon, A. Fuentes-Benites, M. Morales-Rodriguez, B. Jauregui-Rodriguez and E. Cuevas-Yanez, "A facile synthesis of novel miconazole analogues and the evaluation of their antifungal activity," *European journal of medicinal chemistry*, vol. 97, pp. 275-279, 2015.

54. V. Ravichandran and A. Jayakrishnan, "Synthesis and evaluation of anti-fungal activities of sodium alginate-amphotericin B conjugates," *International journal of biological macromolecules*, vol. 108, pp. 1101-1109, 2018.

55. J. d. C. O. Sardi, F. P. Gullo, I. A. Freires, N. d. S. Pitangui, M. P. Segalla, A. M. Fusco-Almeida, P. L. Rosalen, L. O. Regasini and M. J. S. Mendes-Giannini, "Synthesis, antifungal activity of caffeic acid derivative esters, and their synergism with fluconazole and nystatin against Candida spp," *Diagnostic microbiology and infectious disease*, vol. 86, no. 4, pp. 387-391, 2016.

56. R. K. Sharma, V. Singh, N. Tiwari, R. J. Butcher and D. Katiyar, "Synthesis, antimicrobial and chitinase inhibitory activities of 3-amidocoumarins," *Bioorganic Chemistry*, vol. 98, 2020.

57. J. P. Shrestha, C. Baker, Y. Kawasaki, Y. P. Subedi, N. N. Vincent de Paul, J. Y. Takemoto and C.-W. T. Chang, "Synthesis and bioactivity investigation of quinone-based dimeric cationic triazolium amphiphiles selective against resistant fungal and bacterial pathogens," *European journal of medicinal chemistry*, vol. 126, pp. 696-704, 2017.

58. S. K. Shrestha, A. Garzan and S. Garneau-Tsodikova, "Novel alkylated azoles as potent antifungals," *European journal of medicinal chemistry*, vol. 133, pp. 309-318, 2017.

59. L. K. Singh, Priyanka, V. Singh and D. Katiyar, "Design, synthesis and biological evaluation of some new coumarin derivatives as potential antimicrobial agents," *Medicinal chemistry (Shariqah (United Arab Emirates))*, vol. 11, no. 2, pp. 128-134, 2015.

60. D. Stiz, R. Correa, F. D. D'Auria, G. Simonetti and V. Cechinel-Filho, "Synthesis of Cyclic Imides (Methylphtalimides, Carboxylic Acid Phtalimides and Itaconimides) and Evaluation of their Antifungal Potential," *Medicinal chemistry (Shariqah (United Arab Emirates))*, vol. 12, no. 7, pp. 647-654, 2016.

61. Y. P. Subedi, M. N. Alfindee, J. P. Shrestha, G. Becker, M. Grilley, J. Y. Takemoto and C.-W. T. Chang, "Synthesis and biological activity investigation of azole and quinone hybridized phosphonates," *Bioorganic & medicinal chemistry letters*, vol. 28, no. 18, pp. 3034-3037, 2018.

62. D. D. Subhedar, M. H. Shaikh, S. G. Tupe, M. V. Deshpande, V. M. Khedkar, P. C. Jha and B. B. Shingate, "Facile and solvent-free domino synthesis of new quinolidinyl-2,4-thiazolidinones: Antifungal activity and molecular docking," *Mini-Reviews in Medicinal Chemistry*, vol. 18, no. 7, pp. 622-630, 2018.

63. L. Terra, M. Kalil, A. Joffily, H. C. Castro, L. C. E. de, S. S. P. de, T. R. A. Vasconcelos, M. Saito, P. C. Sathler, S. M. V. N. de, C. R. B. Gomes, J. L. Wardell, J. L. Wardell, S. M. S. V. Wardell, G. C. C. Silva and V. O. Lione, "Evaluation of 1,3-benzoxathiol-2-one Derivatives as Potential Antifungal Agents," *Medicinal chemistry (Shariqah (United Arab Emirates))*, vol. 14, no. 3, pp. 304-310, 2018.

64. A. N. Tevyashova, A. M. Korolev, A. S. Trenin, L. G. Dezhenkova, A. A. Shtil, V. I. Polshakov, O. Y. Savelyev and E. N. Olsufyeva, "New conjugates of polyene macrolide amphotericin B with benzoxaboroles: synthesis and properties," *The Journal of antibiotics*, vol. 69, no. 7, pp. 549-560, 2016.

65. N. D. Thanh, H. T. K. Van and T. T. Thu, "Synthesis and characterization of some novel thiosemicarbazones of substituted benzaldehydes and N-(Hepta-O-Acetyl-$\beta{\$}-D-Lactosyl) thiosemicarbazide," *Journal of Carbohydrate Chemistry*, vol. 34, pp. 514-544, 2015.

66. S. V. Tiwari, J. A. Seijas, M. P. Vazquez-Tato, A. P. Sarkate, K. S. Karnik and A. P. G. Nikalje, "Facile Synthesis of Novel Coumarin Derivatives, Antimicrobial Analysis, Enzyme Assay, Docking Study, ADMET Prediction and Toxicity Study," *Molecules (Basel, Switzerland)*, vol. 22, no. 7, 2017.

67. H. Ünver, B. Berber, R. Demirel and A. T. Koparal, "Design, Synthesis, Anti-Proliferative, Anti-microbial, Anti-Angiogenic Activity and In Silico Analysis of Novel Hydrazone Derivatives," *Anti-cancer agents in medicinal chemistry*, vol. 19, no. 13, pp. 1658-1669, 2019.

68. S. e. G. Vasile, M. C. Chifiriuc, C. Kamerzan, L. Marutescu, M. C. Chifiriuc, C. Kamerzan, L. Marutescu, C. Bleotu, C. Kamerzan, C. G. Daniliuc, C. C. Maxim, L. Calu, R. Olar, M. Badea, G. Vasile Scăețeanu, M. C. Chifiriuc, C. Bleotu, C. Kamerzan, L. Măruţescu, C. G. Daniliuc, C. C. Maxim, L. Calu, R. Olar and M. Badea, "Synthesis, Structural Characterization, Antimicrobial Activity, and In Vitro Biocompatibility of New Unsaturated Carboxylate Complexes with 2,2'-Bipyridine," *Molecules (Basel, Switzerland)*, vol. 23, no. 1, 2018.

69. M. M. Victor, R. R. Farias, M. M. Victor, R. R. Farias, S. D. L. da, C. P. H. F. do, R.-S. M. A. de, C. Viegas, P. F. Espuri and M. J. Marques, "Synthesis and Evaluation of Antifungal and Antitrypanosomastid Activities of Symmetrical 1,4-Disubstituted-1,2,3-Bistriazoles Obtained by CuAAC Conditions," *Medicinal chemistry (Shariqah (United Arab Emirates))*, vol. 15, no. 4, pp. 400-408, 2019.

70. D.-J. Wang, Z. Hou, H. Xu, R. An, X. Su and C. Guo, "Design, synthesis, and biological evaluation of 4-chloro-2H-thiochromenes featuring nitrogen-containing side chains as potent antifungal agents," *Bioorganic & medicinal chemistry letters*, vol. 28, no. 22, pp. 3574-3578, 2018.

71. Y. N. Wang, R. R. Y. Bheemanaboina, G. X. Cai and C. H. Zhou, "Novel purine benzimidazoles as antimicrobial agents by regulating ROS generation and targeting clinically resistant Staphylococcus aureus DNA groove," *Bioorganic and Medicinal Chemistry Letters*, vol. 28, no. 9, pp. 1621-1628, 2018.

72. L. Yurttas, Z. A. Kaplancikli, G. Goger and F. Demirci, "Synthesis and anticandidal evaluation of new benzothiazole derivatives with hydrazone moiety," *Journal of enzyme inhibition and medicinal chemistry*, vol. 31, no. 5, pp. 714-720, 2016.

73. J. Zhang, J. Ma, Y. Dong, W. Zhao and J. Feng, "Synthesis and characterization of NH(2)-(AEEA)n-amphotericin B derivatives," *The Journal of antibiotics*, vol. 72, no. 4, pp. 210-217, 2019.

74. Y. Zhang, V. K. R. Tangadanchu, R. R. Y. Bheemanaboina, Y. Cheng and C.-H. Zhou, "Novel carbazole-triazole conjugates as DNA-targeting membrane active potentiators against clinical isolated fungi," *European journal of medicinal chemistry*, vol. 155, pp. 579-589, 2018.

75. D. Zhao, S. Zhao, L. Zhao, X. Zhang, P. Wei, C. Liu, C. Hao, M. Cheng, B. Sun and X. Su, "Discovery of biphenyl imidazole derivatives as potent antifungal agents: Design, synthesis, and structure-activity relationship studies," *Bioorganic & medicinal chemistry*, vol. 25, no. 2, pp. 750-758, 2017.

76. L. Zhao, N. Sun, L. Tian, Y. Sun, Y. Chen, X. Wang, S. Zhao, X. Su, D. Zhao and M. Cheng, "Combating fluconazole-resistant fungi with novel β-azole-phenylacetone derivatives," *European journal of medicinal chemistry*, vol. 183, pp. 111689-111689, 2019.

77. L. Zhao, L. Tian, N. Sun, Y. Sun, Y. Chen, X. Wang, S. Zhao, X. Su, D. Zhao and M. Cheng, "Design, synthesis, and structure-activity relationship studies of l-amino alcohol derivatives as broad-spectrum antifungal agents," *European journal of medicinal chemistry*, vol. 177, pp. 374-385, 2019.

78. S. Zhao, P. Wei, M. Wu, X. Zhang, L. Zhao, X. Jiang, C. Hao, X. Su, D. Zhao and M. Cheng, "Design, synthesis and evaluation of benzoheterocycle analogues as potent antifungal agents targeting CYP51," *Bioorganic & medicinal chemistry*, vol. 26, no. 12, pp. 3242-3253, 2018.

79. S. Zhao, L. Zhao, X. Zhang, C. Liu, C. Hao, H. Xie, M. Cheng, B. Sun and D. Zhao, "Design, synthesis, and structure-activity relationship studies of benzothiazole derivatives as antifungal agents," *European journal of medicinal chemistry*, vol. 123, pp. 514-522, 2016.
